# Biomedical Photoacoustic Imaging and Sensing Using Affordable Resources

**DOI:** 10.3390/s21072572

**Published:** 2021-04-06

**Authors:** Mithun Kuniyil Ajith Singh, Wenfeng Xia

**Affiliations:** 1 Research and Business Development Division, CYBERDYNE INC., Stationsplein 45, A4.004, 3013 AK Rotterdam, The Netherlands; 2 School of Biomedical Engineering & Imaging Sciences, King’s College London, King’s Health Partners, St. Thomas’ Hospital, London SE1 7EH, UK

## 1. Introduction

The photoacoustic (PA) effect, also called the optoacoustic effect, was discovered in the 1880s by Alexander Graham Bell and has been utilized for biomedical imaging and sensing applications since the early 1990s [1]. In biomedical photoacoustic imaging, nanosecond-pulsed or intensity-modulated light illuminates tissue of interest, which when absorbed by intrinsic (such as hemoglobin, lipid) and extrinsic optical absorbers results in the generation of ultrasound (US) signals via thermoelastic expansion. These optically generated US signals can be detected on the tissue surface using conventional ultrasonic probes to generate the tissue’s optical absorption maps with high spatiotemporal resolution. PA imaging thus offers advantages of both US imaging (imaging depth, spatiotemporal resolution) and conventional optical imaging techniques (spectroscopic contrast), making it an ideal modality for structural, functional, and molecular characterization of tissue in situ. Moreover, since both PA and US imaging rely on acoustic detection, it is feasible to share the probe and data acquisition system to perform naturally co-registered dual-mode PA and US imaging with complementary contrast. Since US imaging is ubiquitous in clinics, such a dual-mode approach is expected to facilitate accelerating the clinical translation of the PA imaging technique. Owing to all these advantages, PA imaging has been explored for myriads of preclinical and clinical applications and is undoubtedly one of the fastest-growing biomedical imaging modalities of recent times. Even though PA imaging is matured in lab settings, clinical translation of this promising technique is not happening at an expected pace. One of the important reasons behind this is the costs of pulsed light sources and acoustic detection hardware. Affordability is undoubtedly an important factor to be considered in the following years to help translate PA imaging to clinics around the globe. This first-ever Special Issue focused on biomedical PA imaging and sensing using affordable resources is thus timely, especially considering the fact that this technique is facing an exciting transition from benchtop to bedside. 

The overarching goal of this Special Issue is to provide a current picture of the latest developments in the capabilities of PA imaging and sensing in an affordable setting, such as advances in the technology involving light sources, and delivery, acoustic detection, and image reconstruction and processing algorithms. This issue includes 13 papers (2 reviews, 2 letters, 1 communication and 8 full-length articles) which cover a comprehensive spectrum of research from technology developments and novel imaging methods to preclinical and clinical studies, predominantly in a cost-effective setting.

## 2. Review Articles

The Issue starts with a comprehensive review article on portable and affordable light-source-based PA tomography from Kuniyil Ajith Singh and Xia [2]. In this review, the authors focus on (1) basics of PA imaging, (2) cost-effective pulsed light sources for PA imaging and (3) important preclinical and clinical applications reported until now using affordable-light-source-based PA imaging. Because of tremendous developments in solid-state device technology, high-power LEDs have been explored heavily in recent years as illumination sources in PA imaging. In another interesting paper, for the first time, Zhu et al. comprehensively reviewed the use of LEDs in biomedical PA imaging, covering all technical details, preclinical and clinical applications reported until now [3].

## 3. Original Papers—Full-Length Articles, Communications, and Letters

The rest of the original papers in this issue are summarized in the following sub-sections based on the contents.

### 3.1. Instrumentation, Technology Developments

Conventional US probes are opaque and are not ideal for miniaturized PA imaging systems, especially for microscopic applications. In an exciting work from Chen et al., the authors developed an affordable transparent lithium niobate (LiNbO_3_) US transducer with a 13-MHz central frequency and a 60% reception bandwidth for optical resolution PA microscopy [4]. The authors tested the system performance by imaging vasculature in chicken embryos and melanoma depth profiling using tissue phantoms. The system proposed in this work is expected to have a promising future in wearable and high-throughput imaging applications.

LED-based PA imaging is gaining more popularity in recent years because of its portability, affordability, and ease of use. However, due to the large beam divergence of LEDs compared to traditional laser beams, it is of paramount importance to quantify the angular dependence of LED-based illumination and optimize its performance for imaging superficial or deep-seated lesions. Kuriakose et al. reported on the development of a custom 3D printed LED array holder to be used along with a commercial LED-based PA imaging system (Acoustic X, CYBERDYNE INC, Tsukuba, Japan) and demonstrated the importance of changing LED illumination angle when used for superficial and deep-tissue applications [5].

Considering the tradeoffs between portability, cost, optical energy, and frame rate, it is critical to compare the PA imaging performance of LED and laser illuminations to help select a suitable source for a given biomedical imaging application. Agrawal et al. reported on the development of a setup for a head-to-head comparison of LED and laser-based PA imaging of vasculature [6]. With measurements on tissue-mimicking phantoms and human volunteers, authors concluded that LED-based PA imaging performs equally and sometimes even better than laser-based systems demonstrating its strong potential to be a mobile health care technology for diagnosing vascular diseases such as peripheral arterial disease and stroke in point-of-care and resource-limited settings.

In applications like wound screening and laser surgery guidance, conventional PA imaging systems that usually require US probes in contact with the tissue are not an ideal option. In a work by Lengenfelder et al., it is demonstrated for the first time that remote PA sensing by speckle analysis can be performed in the MHz sampling range by tracking a single speckle using a four-quadrant photo-detector [7]. By demonstrating PA sensing and endoscopic capabilities, this work demonstrated that single speckle sensing is, therefore, an easy, robust, contact-free photoacoustic detection technique and holds the potential for economical, and ultra-fast PA sensing.

### 3.2. Image Processing and Enhancement Techniques

Multispectral PA imaging can be used to non-invasively visualize and quantify tissue chromophores with high spatial resolution. Utilizing multiwavelength PA data, one can characterize the spectral absorption signature of prominent tissue chromophores, such as hemoglobin or lipid, by using spectral unmixing methods. Grasso et al. reported the feasibility of an unsupervised spectral unmixing algorithm to detect and extract the tissue chromophores without any a priori knowledge of the optical absorption spectra of the tissue chromophores or exogenous contrast agents and user interactions [8]. The authors validated this novel algorithm using simulations, phantom studies, and mouse in vivo experiments and demonstrated the feasibility of extracting and quantify the tissue chromophores in a completely unsupervised manner. 

Conventionally, hand-held linear-array-based PA imaging systems operate in the reflection mode using a dark-field illumination scheme, where the illumination is on both sides of the elevation plane (short-axis) of the US probe. Uliana et al. reported and demonstrated a novel multiangle long-axis lateral illumination approach with several advantages [9]. Phantom, animal, and human in vivo results in this work demonstrate a remarkable improvement of the new illumination approach in light delivery for targets with a width smaller than the transducer’s lateral dimension.

Portable and affordable light sources like pulsed laser diodes and LEDs have the potential in accelerating the clinical translation of PA imaging. However, pulse energy offered by these sources is often low when compared to solid-state lasers, thus resulting in a low signal-to-noise-ratio (SNR), especially in deep-tissue applications. Improvement of the SNR is of paramount importance in these cases. Thomas et al. proposed a continuous-wave laser-based pre-illumination approach to increase the temperature of the imaging sample and thus the PA signal strength from it [10]. In this work, using tissue-mimicking phantoms and common contrast agents, the authors showed the feasibility of enhancing the PA signal strength significantly. 

### 3.3. Preclinical and Clinical Applications

Small animals are widely used as disease models in medical research, especially in the pharmaceutical industry. Since PA imaging can offer functional and molecular information, it is an ideal modality for small animal imaging. Kalloor Joseph et al. reported a portable and affordable approach for performing fast tomographic PA and US imaging of small animals [11]. In this work, the authors used LED-based PA and US tomographic imaging and showed its potential in liver fibrosis research. 

Conventional PA imaging systems utilize expensive and bulky solid-state lasers with low pulse repetition rates; as such, their availability for preclinical cancer research is hampered. In an interesting study from Xavierselvan et al., the authors validated the capability of an LED-based PA and US imaging system for monitoring heterogeneous microvasculature in tumors (up to 10 mm in depth) and quantitatively compared the PA images with gold standard histology images [12]. The results of this work give a direct confirmation that LED-based PA and US imaging hold the potential to be a valuable tool in preclinical cancer research.

Hypoxia and hyper-vascularization are the hallmarks of cancer and oxygen saturation imaging is arguably the most important application of PA imaging. By illuminating tissue using two wavelengths, it is feasible to probe the oxygen saturation of tissue with microvasculature scale resolution. Bulsink et al. reported fluence-compensated oxygen saturation imaging using two wavelength LED-based PA imaging [13]. In this work, the authors demonstrated real-time fluence compensated oxygen saturation imaging in phantoms, small animals, and measurements on human volunteers.

Follicular unit extraction and follicular unit transplantation are used in most hair transplant procedures. In both cases, it is important for clinicians to characterize follicle density for treatment planning and evaluation. Hariri et al. utilized 2D and 3D LED-based PA imaging for measuring follicle density and angles across regions of varying density [14]. The authors validated the idea using experiments on small animals and also using measurements on healthy human volunteers. 

## 4. Summary

PA imaging is growing at a tremendous pace and is expected to reach clinics soon. At this point, this promising technology is facing an exciting transition phase and thus this Special Issue with a focus on affordability is timely. Thirteen excellent papers in this Special Issue from academia and industry represent a small sample that demonstrates the immense developments in the field. We hope that this Special Issue can provide the motivation and inspiration for further technological advances in this exciting field and accelerates the clinical translation of this promising biomedical imaging modality. 
